# INTERACTIONS OF OPTIMISM WITH SOCIAL RELATIONS FOR WOMEN'S WELL-BEING

**DOI:** 10.1093/geroni/igz038.776

**Published:** 2019-11-08

**Authors:** Aurora M Sherman

**Affiliations:** Oregon State University, Corvallis, Oregon, United States

## Abstract

The impact of personality on the relationship between social relations and well-being has been understudied. We assessed optimism, social support, and social strain in association with self-esteem, depressive symptoms and life satisfaction for a sample of 247 women (Mean age = 57.56, range 45-89 years) from three race groups (42% Native American, 34% African American, 24% European American). PROCESS models revealed significant interactions between optimism and support suggesting that high support buffers the risk of low optimism for all three dependent variables, and two interactions of optimism with social strain, showing that low optimism exacerbated the negative impact of high strain for CES-D and self-esteem scores. The full models accounted for 30-50% of the variance explained in each outcome. We discuss important resources for resilience shown by the women in the sample.

